# ECRG-4 expression in normal and neoplastic choroid plexus

**DOI:** 10.1186/1743-8454-7-S1-S32

**Published:** 2010-12-15

**Authors:** John E Donahue, Miles C Miller, Virginia Breese, Sonia Podvin, Brian Eliceiri, Cynthia L Jackson, Conrad E Johanson, Edward G Stopa, Ana Maria Gonzalez, Andrew Baird

**Affiliations:** 1 Dept. of Pathology, Rhode Island Hospital/Alpert Medical School, 593 Eddy St (APC 12115), Providence, RI 02903, USA; 2 Dept. of Surgery, UCSD Medical Center, 212 Dickinson St, CTFB310 MS 8296, San Diego, CA 92103, USA; 3 Dept. of Neurosurgery, Rhode Island Hospital/Alpert Medical School, 593 Eddy St, Providence, RI 02903, USA; 4 Neuropharmacology and Neurobiology Section, School of Clinical and Experimental Medicine, College of Medical and Dental Sciences, Institute of Biomedical Research (West), University of Birmingham, Edgbaston, Birmingham B15 2TT, UK

## Background

The choroid plexus is a major site of gene expression of esophageal cancer-related gene (ECRG)-4 during development, suggesting that its gene product may be involved in cerebrospinal fluid (CSF) homeostasis. Yet, ECRG-4 is also a novel candidate tumor suppressor gene whose expression is downregulated and is inversely associated with a worse prognosis in several different cancers. Reduced expression of ECRG-4 has been demonstrated in most tumors, including colorectal carcinoma and malignant glioma, to be mediated by hypermethylation of its promoter.

## Materials and methods

In this study, samples of normal human choroid plexus (both fetal and adult) and choroid plexus neoplasms (WHO grade I papilloma, grade II atypical papilloma, and grade III carcinoma) were stained with antibodies that we generated to augurin, the gene product of ECRG-4. DNA was then extracted from the tissue, treated with bisulfite, and subjected to PCR using a 217-base pair region encompassing the ECRG-4 promotor to detect methylation.

## Results

Both fetal and adult human choroid plexus cells demonstrated a robust positive immunostaining at the apical surface that is consistent with our prior results in human, rat, and mouse brains. In contrast, there was a near-complete absence of immunostaining in all of the choroid plexus neoplasms examined. The choroid plexus carcinoma demonstrated significant methylation of the ECRG-4 promotor region.

## Conclusions

Taken together, these data suggest that ECRG-4 is downregulated in neoplasms of the choroid plexus just as has been observed in other central nervous system (CNS) and non-CNS cancers. This is likely due to hypermethylation of the ECRG-4 promotor, as shown in the choroid plexus carcinoma. Further analysis is underway to determine the (1) physiologic and (2) pathophysiologic consequences of ECRG-4 over- and under- expression in the choroid plexus on CSF formation, function, and composition.

